# 1-{(*E*)-[5-(2-Nitro­phen­yl)furan-2-yl]methyl­idene}-2,2-diphenyl­hydrazine

**DOI:** 10.1107/S1600536812050246

**Published:** 2012-12-15

**Authors:** Marcos Flores-Alamo, Blanca M. Cabrera-Vivas, Ruth Meléndrez-Luevano, Julio M. Hernández P., Lena Ruiz-Azuara

**Affiliations:** a Facultad de Química, Universidad Nacional Autónoma de México, 04510, México DF, Mexico; b Facultad de Ciencias Químicas, Benemérita Universidad Autónoma de Puebla 72570, Puebla, Pue., Mexico

## Abstract

In the title compound, C_23_H_17_N_3_O_3_, the terminal benzene rings are oriented at dihedral angles of 3.67 (7), 76.02 (7) and 16.37 (7)° with respect to the central furan ring. In the crystal, mol­ecules are connected *via* weak C—H⋯O hydrogen bonds, resulting in a three-dimensional supra­molecular array.

## Related literature

For applications of hydrazones, see: Robinson (1963 ▶); Sztanke *et al.* (2007 ▶); Al-Macrosaur *et al.* (2007 ▶); Kucukguzel *et al.* (2003 ▶); Roma *et al.* (2000 ▶); Smalley *et al.* (2006 ▶); Gemma *et al.* (2006 ▶). For hydrogen-bond motifs, see: Etter *et al.* (1990 ▶).
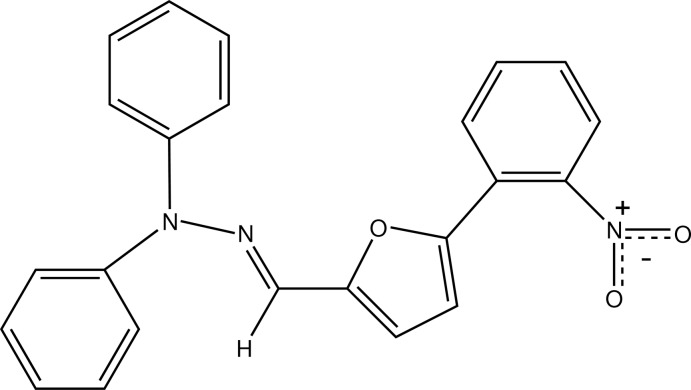


## Experimental

### 

#### Crystal data

C_23_H_17_N_3_O_3_
*M* *_r_* = 383.4
Monoclinic, 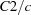

*a* = 11.2439 (2) Å
*b* = 17.3325 (4) Å
*c* = 19.7575 (4) Å
β = 105.778 (2)°
*V* = 3705.36 (13) Å^3^
*Z* = 8
Cu *K*α radiation
μ = 0.76 mm^−1^
*T* = 130 K
0.58 × 0.23 × 0.16 mm

#### Data collection

Oxford Diffraction Xcalibur (Atlas, Gemini) diffractometer
Absorption correction: analytical (*CrysAlis RED*; Oxford Diffraction, 2009 ▶) *T*_min_ = 0.759, *T*_max_ = 0.892
12881 measured reflections
3395 independent reflections
3070 reflections with *I* > 2σ(*I*)
*R*_int_ = 0.025

#### Refinement

*R*[*F* ^2^ > 2σ(*F* ^2^)] = 0.037
*wR*(*F* ^2^) = 0.097
*S* = 1.03
3395 reflections
262 parameters
H-atom parameters constrained
Δρ_max_ = 0.15 e Å^−3^
Δρ_min_ = −0.23 e Å^−3^

### 

Data collection: *CrysAlis CCD* (Oxford Diffraction, 2009 ▶); cell refinement: *CrysAlis RED* (Oxford Diffraction, 2009 ▶); data reduction: *CrysAlis RED*; program(s) used to solve structure: *SHELXS97* (Sheldrick, 2008 ▶); program(s) used to refine structure: *SHELXL97* (Sheldrick, 2008 ▶); molecular graphics: *ORTEP-3 for Windows* (Farrugia, 2012 ▶); software used to prepare material for publication: *WinGX* (Farrugia, 2012 ▶).

## Supplementary Material

Crystal structure: contains datablock(s) global, I. DOI: 10.1107/S1600536812050246/xu5663sup1.cif

Structure factors: contains datablock(s) I. DOI: 10.1107/S1600536812050246/xu5663Isup2.hkl

Supplementary material file. DOI: 10.1107/S1600536812050246/xu5663Isup3.cml

Additional supplementary materials:  crystallographic information; 3D view; checkCIF report

## Figures and Tables

**Table 1:** Hydrogen-bond geometry (Å, °)

*D*—H⋯*A*	*D*—H	H⋯*A*	*D*⋯*A*	*D*—H⋯*A*
C8—H8⋯O2^i^	0.95	2.48	3.1294 (16)	126
C11—H11⋯O3^ii^	0.95	2.57	3.4336 (18)	151
C12—H12⋯O3^iii^	0.95	2.48	3.3786 (18)	158

Symmetry codes: (i) 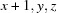; (ii) 
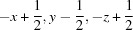
; (iii) 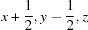.

**Fig. 1.**
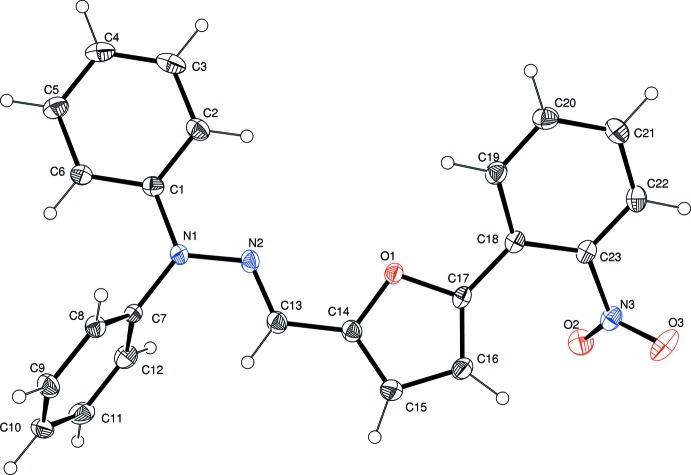
The structure of the title compound, showing the atomic numbering scheme. Non-H atoms are shown with 50% probability displacement ellipsoids.

## supplementary crystallographic information

### Comment

Hydrazones are nitrogenated derivatives of carbonyl groups. Their general structure contains a double carbon-nitrogen bond formed by the elimination of a water molecule when it reacts with a hydrazine having a carbonyl compound. Many hydrazones, including diphenylhydrazones, have several industrial purposes such as hole carriers in thin film organic photoconductors applied to electrographic processes in printers and photocopiers, plasticizers, polymer stabilizers, antioxidants and polymer initiators (Robinson, 1963). Moreover, hydrazides and hydrazones are present in many of the bioactive heterocyclic compounds because of their diverse biological and clinical applications, making them of great interest for researchers who have synthesized a variety of hydrazide-hydrazones derivatives and have screened them for their various biological activities anticancerigenous (Sztanke *et al.*, 2007), anti-HIV (Al-Macrosaur *et al.*, 2007), antimycobacterial (Kucukguzel *et al.*, 2003), anti-inflammatory, antidiabetic, antimicrobial, and antimalarial activities (Roma *et al.*, 2000; Smalley *et al.*, 2006; Gemma *et al.*, 2006).

In the title compound C_23_H_17_N_3_O_3_, the asymmetric unit consist of one molecule of [5-(2-nitrophenyl)furan-2-ylmethylene]-2,2-diphenylhydrazine (Fig. 1) showing an E configuration on C=N group with diphenylhydrazine group opposite to nitrophenylfuran group. The terminal benzene rings are oriented with respect to the central furan ring at 3.67 (7), 76.02 (7) and 16.37 (7)°, respectively. The angle between planes formed by phenyl rings C1 to C6 (r.m.s. = 0.0054) and C7 to C12 (*r.m.s.* = 0.0045) is 74.46 (4) °. The furan-2-ylmethylene fragment shows slight planary deviation with *r.m.s.* of 0.0153 and plane equation 6.262 (3) *x* + 14.321 (4) *y* - 4.589 (11) *z* = 4.215 (5), while in the *o*-nitrophenyl group, the angle (53.76 (8)°) between planes NO_2_ and phenyl ring and the *r.m.s.* of 0.3714 evidence a deviation of planary.

The conformation of nitro group with respect to phenyl ring is favoured by the intermolecular interactions C—H···O of type hydrogen bond (table 1). The intermolecular contacts C8—H8···O2 and C9—H9···O2 toits neighbours related by the symmetry operation *x* + 1, *y*, *z* showing a *R_2_^1^(5)* motif (Etter *et al.*, 1990) and formed a chain in the direction of the crystallographic *a* axis, while the C11—H11···O1 and C12—H12···O1 with symmetry operations -*x* + 1/2, *y* - 1/2, -*z* + 1/2 and *x* + 1/2, *y* - 1/2, *z* respectively show a motif of the type D mainly. All intermolecular interactions are observed growing along the *a*, *b* and *c* axes, resulting in a three-dimensional supramolecular array.

### Experimental

Diphenylhydrazine (1.38 mmol, 254 mg) was dissolved in ethanol, a chemical equivalent (300 mg) of aldehyde which was previously dissolved in the same solvent and it was added drop by drop stirring constantly.The action mixture was kept at room temperature and was monitored by TLC, and then vacuum filtered. The hydrazones were recrystallized by a continuous and controlled process until wine crystals with adequate size and purity were developed in order to obtain X-ray studies. m.p. = 393-395 K, Yield 90.6%.

^1^H NMR (400 MHz, (CD_3_)_2_CO: (δ/ p.p.m., J/Hz): 7.77 (dd, H-3, *J* =7.94 H3 –H4 *J* =1.20 coupling W H3 –H5); 7.64 (dd, H-6, *J* =8.10 H6 –H5 and *J* =1.16 coupling W H6 –H4); 7.54 (td, H-5, coupling H-4 H-6 *J* =7.74 and coupling W H-3, *J* =1.20); 7.42 (t, H-3); 7.36 (td, H-4 coupling H-3 H-5, *J* = 7.74, coupling W H-6, *J* = 1.36); 7.20 (m, 4H,H-2, H2,H-4); 6.99 (s, H-i); 6.69 (d, H-4 coupling H-3, *J* =3.60); 6.58 (d, H-3 coupling H-4, *J* = 3.60). ^13^C NMR (100 MHz, (CD_3_)_2_CO): (δ/ p.p.m.):153.0 (C2), 147.56 (C5), 147.17 (C2), 142.92 (C1), 131.77 (C5), 129.84 (C3), 128.50 (C3), 127.97 (C4), 124.87 (C2), 124.79 (iminic-C), 123.81 (C1), 123.69 (C6), 122.40 (C4), 111.91 (C4) *y* 110.10 (C3).

### Refinement

H atoms bonded to C atoms were placed in geometrical idealized positions and were refined as riding on their parent atoms, with C—H = 0.95 Å with *U*_iso_(H) = 1.2 *U*_eq_(C).

### Crystal data

**Table:**

C_23_H_17_N_3_O_3_	*F*(000) = 1600
*M**_r_* = 383.4	*D*_x_ = 1.375 Mg m^−^^3^
Monoclinic, *C*2/*c*	Cu *K*α radiation, λ = 1.54184 Å
*a* = 11.2439 (2) Å	Cell parameters from 7112 reflections
*b* = 17.3325 (4) Å	θ = 4.7–68.0°
*c* = 19.7575 (4) Å	µ = 0.76 mm^−^^1^
β = 105.778 (2)°	*T* = 130 K
*V* = 3705.36 (13) Å^3^	Prism, dark red
*Z* = 8	0.58 × 0.23 × 0.16 mm

### Data collection

**Table:**

Oxford Diffraction Xcalibur (Atlas, Gemini) diffractometer	3395 independent reflections
Graphite monochromator	3070 reflections with *I* > 2σ(*I*)
Detector resolution: 10.4685 pixels mm^-1^	*R*_int_ = 0.025
ω scans	θ_max_ = 68.1°, θ_min_ = 4.7°
Absorption correction: analytical (*CrysAlis RED*; Oxford Diffraction, 2009)	*h* = −13→12
*T*_min_ = 0.759, *T*_max_ = 0.892	*k* = −20→20
12881 measured reflections	*l* = −23→21

### Refinement

**Table:**

Refinement on *F*^2^	Primary atom site location: structure-invariant direct methods
Least-squares matrix: full	Secondary atom site location: difference Fourier map
*R*[*F*^2^ > 2σ(*F*^2^)] = 0.037	Hydrogen site location: inferred from neighbouring sites
*wR*(*F*^2^) = 0.097	H-atom parameters constrained
*S* = 1.03	*w* = 1/[σ^2^(*F*_o_^2^) + (0.0507*P*)^2^ + 2.4695*P*] where *P* = (*F*_o_^2^ + 2*F*_c_^2^)/3
3395 reflections	(Δ/σ)_max_ < 0.001
262 parameters	Δρ_max_ = 0.15 e Å^−^^3^
0 restraints	Δρ_min_ = −0.23 e Å^−^^3^

### Special details

**Table:**

Geometry. All s.u.'s (except the s.u. in the dihedral angle between two l.s. planes) are estimated using the full covariance matrix. The cell s.u.'s are taken into account individually in the estimation of s.u.'s in distances, angles and torsion angles; correlations between s.u.'s in cell parameters are only used when they are defined by crystal symmetry. An approximate (isotropic) treatment of cell s.u.'s is used for estimating s.u.'s involving l.s. planes.
Refinement. Refinement of *F*^2^ against ALL reflections. The weighted *R*-factor *wR* and goodness of fit *S* are based on *F*^2^, conventional *R*-factors *R* are based on *F*, with *F* set to zero for negative *F*^2^. The threshold expression of *F*^2^ > 2σ(*F*^2^) is used only for calculating *R*-factors(gt) *etc*. and is not relevant to the choice of reflections for refinement. *R*-factors based on *F*^2^ are statistically about twice as large as those based on *F*, and *R*- factors based on ALL data will be even larger.

### Fractional atomic coordinates and isotropic or equivalent isotropic displacement parameters (Å^2^)

**Table:**

	*x*	*y*	*z*	*U*_iso_*/*U*_eq_	
C1	0.78362 (12)	0.11751 (7)	0.50986 (6)	0.0257 (3)	
C2	0.74740 (13)	0.14665 (8)	0.56708 (7)	0.0325 (3)	
H2	0.672	0.1743	0.5597	0.039*	
C3	0.82184 (16)	0.13506 (9)	0.63460 (8)	0.0413 (4)	
H3	0.796	0.154	0.6734	0.05*	
C4	0.93315 (15)	0.09646 (9)	0.64657 (7)	0.0409 (4)	
H4	0.9841	0.0896	0.6931	0.049*	
C5	0.96930 (14)	0.06805 (9)	0.59025 (7)	0.0357 (3)	
H5	1.0458	0.0416	0.5981	0.043*	
C6	0.89522 (12)	0.07768 (8)	0.52221 (7)	0.0300 (3)	
H6	0.9206	0.0571	0.4838	0.036*	
C7	0.74945 (11)	0.10332 (7)	0.38131 (6)	0.0241 (3)	
C8	0.84143 (12)	0.14515 (7)	0.36340 (7)	0.0275 (3)	
H8	0.8763	0.1892	0.3901	0.033*	
C9	0.88229 (13)	0.12232 (9)	0.30637 (7)	0.0349 (3)	
H9	0.946	0.1505	0.2943	0.042*	
C10	0.83115 (14)	0.05903 (9)	0.26718 (7)	0.0374 (3)	
H10	0.8599	0.0435	0.2283	0.045*	
C11	0.73814 (15)	0.01799 (9)	0.28417 (8)	0.0401 (4)	
H11	0.7021	−0.0252	0.2566	0.048*	
C12	0.69702 (13)	0.03992 (8)	0.34186 (7)	0.0339 (3)	
H12	0.6335	0.0115	0.354	0.041*	
C13	0.53250 (12)	0.18208 (8)	0.37108 (7)	0.0289 (3)	
H13	0.5551	0.1629	0.3312	0.035*	
C14	0.42085 (12)	0.22646 (7)	0.36118 (7)	0.0277 (3)	
C15	0.33253 (12)	0.24501 (8)	0.30155 (7)	0.0320 (3)	
H15	0.3308	0.2304	0.2549	0.038*	
C16	0.24368 (12)	0.29027 (8)	0.32204 (7)	0.0318 (3)	
H16	0.1708	0.312	0.2919	0.038*	
C17	0.28245 (11)	0.29681 (7)	0.39320 (7)	0.0259 (3)	
C18	0.24109 (11)	0.33793 (7)	0.44712 (7)	0.0251 (3)	
C19	0.32397 (12)	0.35087 (8)	0.51312 (7)	0.0307 (3)	
H19	0.4049	0.3299	0.5228	0.037*	
C20	0.29179 (13)	0.39311 (8)	0.56460 (7)	0.0352 (3)	
H20	0.3503	0.4001	0.609	0.042*	
C21	0.17507 (14)	0.42559 (8)	0.55249 (8)	0.0352 (3)	
H21	0.1538	0.4553	0.5879	0.042*	
C22	0.09046 (12)	0.41394 (7)	0.48811 (8)	0.0311 (3)	
H22	0.0101	0.4358	0.4787	0.037*	
C23	0.12323 (11)	0.37034 (7)	0.43747 (7)	0.0260 (3)	
O2	−0.00013 (9)	0.29075 (6)	0.35265 (6)	0.0402 (3)	
O3	−0.03133 (10)	0.41347 (7)	0.34252 (6)	0.0497 (3)	
N1	0.70834 (10)	0.12687 (7)	0.44078 (6)	0.0295 (3)	
N2	0.60193 (10)	0.16807 (6)	0.43303 (6)	0.0281 (2)	
N3	0.02437 (10)	0.35707 (7)	0.37240 (6)	0.0304 (3)	
O1	0.39230 (7)	0.25773 (5)	0.41814 (4)	0.0260 (2)	

### Atomic displacement parameters (Å^2^)

**Table:**

	*U*^11^	*U*^22^	*U*^33^	*U*^12^	*U*^13^	*U*^23^
C1	0.0271 (7)	0.0272 (6)	0.0240 (6)	−0.0049 (5)	0.0089 (5)	0.0014 (5)
C2	0.0336 (7)	0.0356 (7)	0.0318 (7)	−0.0052 (6)	0.0147 (6)	−0.0051 (6)
C3	0.0538 (10)	0.0468 (9)	0.0273 (7)	−0.0110 (7)	0.0177 (7)	−0.0089 (6)
C4	0.0469 (9)	0.0488 (9)	0.0232 (7)	−0.0084 (7)	0.0032 (6)	0.0009 (6)
C5	0.0357 (8)	0.0394 (8)	0.0291 (7)	−0.0008 (6)	0.0040 (6)	0.0050 (6)
C6	0.0309 (7)	0.0349 (7)	0.0248 (6)	0.0013 (5)	0.0089 (5)	0.0012 (5)
C7	0.0206 (6)	0.0300 (6)	0.0213 (6)	0.0034 (5)	0.0052 (5)	0.0028 (5)
C8	0.0253 (6)	0.0293 (7)	0.0284 (6)	0.0004 (5)	0.0082 (5)	0.0003 (5)
C9	0.0347 (7)	0.0426 (8)	0.0327 (7)	0.0062 (6)	0.0179 (6)	0.0075 (6)
C10	0.0460 (8)	0.0447 (8)	0.0228 (7)	0.0175 (7)	0.0118 (6)	0.0037 (6)
C11	0.0470 (9)	0.0340 (8)	0.0320 (7)	0.0055 (6)	−0.0018 (6)	−0.0087 (6)
C12	0.0299 (7)	0.0325 (7)	0.0372 (7)	−0.0033 (6)	0.0054 (6)	0.0010 (6)
C13	0.0266 (7)	0.0331 (7)	0.0302 (7)	0.0003 (5)	0.0130 (5)	0.0030 (5)
C14	0.0256 (6)	0.0303 (7)	0.0293 (6)	−0.0001 (5)	0.0112 (5)	0.0019 (5)
C15	0.0308 (7)	0.0386 (7)	0.0272 (7)	0.0013 (6)	0.0086 (5)	0.0004 (5)
C16	0.0259 (7)	0.0382 (7)	0.0293 (7)	0.0038 (5)	0.0042 (5)	0.0038 (5)
C17	0.0193 (6)	0.0269 (6)	0.0301 (7)	0.0020 (5)	0.0044 (5)	0.0047 (5)
C18	0.0219 (6)	0.0236 (6)	0.0294 (6)	−0.0014 (5)	0.0065 (5)	0.0040 (5)
C19	0.0239 (6)	0.0352 (7)	0.0317 (7)	0.0000 (5)	0.0055 (5)	0.0012 (5)
C20	0.0339 (7)	0.0386 (8)	0.0314 (7)	−0.0058 (6)	0.0062 (6)	−0.0040 (6)
C21	0.0384 (8)	0.0309 (7)	0.0400 (8)	−0.0036 (6)	0.0171 (6)	−0.0056 (6)
C22	0.0269 (7)	0.0256 (6)	0.0429 (8)	0.0005 (5)	0.0130 (6)	0.0022 (6)
C23	0.0218 (6)	0.0226 (6)	0.0328 (7)	−0.0024 (5)	0.0060 (5)	0.0044 (5)
O2	0.0311 (5)	0.0397 (6)	0.0470 (6)	−0.0091 (4)	0.0057 (4)	−0.0080 (5)
O3	0.0318 (6)	0.0470 (6)	0.0595 (7)	0.0004 (5)	−0.0060 (5)	0.0201 (5)
N1	0.0242 (5)	0.0414 (6)	0.0251 (6)	0.0067 (5)	0.0103 (4)	0.0022 (5)
N2	0.0222 (5)	0.0328 (6)	0.0320 (6)	0.0025 (4)	0.0121 (4)	0.0039 (4)
N3	0.0195 (5)	0.0346 (6)	0.0367 (6)	−0.0019 (4)	0.0068 (5)	0.0060 (5)
O1	0.0208 (4)	0.0298 (5)	0.0271 (5)	0.0031 (3)	0.0060 (3)	0.0029 (3)

### Geometric parameters (Å, º)

**Table:**

C1—C6	1.3943 (19)	C14—C15	1.3569 (19)
C1—C2	1.3961 (18)	C14—O1	1.3638 (15)
C1—N1	1.4062 (16)	C14—O1	1.3638 (15)
C2—C3	1.383 (2)	C15—C16	1.4133 (19)
C2—H2	0.95	C15—H15	0.95
C3—C4	1.382 (2)	C16—C17	1.3587 (19)
C3—H3	0.95	C16—H16	0.95
C4—C5	1.375 (2)	C17—O1	1.3770 (14)
C4—H4	0.95	C17—O1	1.3770 (14)
C5—C6	1.3856 (19)	C17—C18	1.4590 (18)
C5—H5	0.95	C18—C19	1.3994 (18)
C6—H6	0.95	C18—C23	1.4039 (18)
C7—C12	1.3830 (19)	C19—C20	1.379 (2)
C7—C8	1.3855 (18)	C19—H19	0.95
C7—N1	1.4340 (16)	C20—C21	1.388 (2)
C8—C9	1.3852 (18)	C20—H20	0.95
C8—H8	0.95	C21—C22	1.380 (2)
C9—C10	1.375 (2)	C21—H21	0.95
C9—H9	0.95	C22—C23	1.3814 (19)
C10—C11	1.380 (2)	C22—H22	0.95
C10—H10	0.95	C23—N3	1.4711 (17)
C11—C12	1.394 (2)	O2—O2	0
C11—H11	0.95	O2—N3	1.2212 (15)
C12—H12	0.95	O3—N3	1.2228 (15)
C13—N2	1.2833 (17)	N1—N2	1.3657 (15)
C13—C14	1.4396 (18)	N3—O2	1.2212 (15)
C13—H13	0.95	O1—O1	0.000 (3)
			
C6—C1—C2	118.96 (12)	C14—C15—C16	106.87 (12)
C6—C1—N1	120.17 (11)	C14—C15—H15	126.6
C2—C1—N1	120.86 (12)	C16—C15—H15	126.6
C3—C2—C1	119.68 (14)	C17—C16—C15	106.84 (12)
C3—C2—H2	120.2	C17—C16—H16	126.6
C1—C2—H2	120.2	C15—C16—H16	126.6
C4—C3—C2	121.18 (13)	C16—C17—O1	109.49 (11)
C4—C3—H3	119.4	C16—C17—O1	109.49 (11)
C2—C3—H3	119.4	O1—C17—O1	0.00 (9)
C5—C4—C3	119.22 (13)	C16—C17—C18	136.05 (12)
C5—C4—H4	120.4	O1—C17—C18	114.32 (10)
C3—C4—H4	120.4	O1—C17—C18	114.32 (10)
C4—C5—C6	120.65 (14)	C19—C18—C23	115.29 (12)
C4—C5—H5	119.7	C19—C18—C17	119.67 (11)
C6—C5—H5	119.7	C23—C18—C17	124.95 (11)
C5—C6—C1	120.29 (12)	C20—C19—C18	122.04 (13)
C5—C6—H6	119.9	C20—C19—H19	119
C1—C6—H6	119.9	C18—C19—H19	119
C12—C7—C8	120.34 (12)	C19—C20—C21	120.91 (13)
C12—C7—N1	120.33 (12)	C19—C20—H20	119.5
C8—C7—N1	119.32 (11)	C21—C20—H20	119.5
C9—C8—C7	119.61 (12)	C22—C21—C20	118.82 (13)
C9—C8—H8	120.2	C22—C21—H21	120.6
C7—C8—H8	120.2	C20—C21—H21	120.6
C10—C9—C8	120.36 (13)	C21—C22—C23	119.64 (12)
C10—C9—H9	119.8	C21—C22—H22	120.2
C8—C9—H9	119.8	C23—C22—H22	120.2
C9—C10—C11	120.18 (13)	C22—C23—C18	123.26 (12)
C9—C10—H10	119.9	C22—C23—N3	115.56 (11)
C11—C10—H10	119.9	C18—C23—N3	121.14 (11)
C10—C11—C12	120.01 (13)	O2—O2—N3	0 (10)
C10—C11—H11	120	N2—N1—C1	116.55 (10)
C12—C11—H11	120	N2—N1—C7	121.65 (10)
C7—C12—C11	119.48 (13)	C1—N1—C7	121.28 (10)
C7—C12—H12	120.3	C13—N2—N1	119.54 (11)
C11—C12—H12	120.3	O2—N3—O2	0.00 (15)
N2—C13—C14	120.83 (12)	O2—N3—O3	123.84 (12)
N2—C13—H13	119.6	O2—N3—O3	123.84 (12)
C14—C13—H13	119.6	O2—N3—C23	118.53 (11)
C15—C14—O1	109.97 (11)	O2—N3—C23	118.53 (11)
C15—C14—O1	109.97 (11)	O3—N3—C23	117.59 (11)
O1—C14—O1	0.00 (6)	O1—O1—C14	0 (10)
C15—C14—C13	130.53 (12)	O1—O1—C17	0 (10)
O1—C14—C13	119.50 (11)	C14—O1—C17	106.83 (9)
O1—C14—C13	119.50 (11)		
			
C6—C1—C2—C3	−0.5 (2)	C20—C21—C22—C23	0.2 (2)
N1—C1—C2—C3	178.48 (12)	C21—C22—C23—C18	−1.6 (2)
C1—C2—C3—C4	1.4 (2)	C21—C22—C23—N3	176.22 (12)
C2—C3—C4—C5	−1.1 (2)	C19—C18—C23—C22	1.75 (18)
C3—C4—C5—C6	−0.2 (2)	C17—C18—C23—C22	−174.89 (12)
C4—C5—C6—C1	1.0 (2)	C19—C18—C23—N3	−175.97 (11)
C2—C1—C6—C5	−0.7 (2)	C17—C18—C23—N3	7.40 (19)
N1—C1—C6—C5	−179.69 (12)	C6—C1—N1—N2	−178.57 (11)
C12—C7—C8—C9	−1.14 (19)	C2—C1—N1—N2	2.45 (18)
N1—C7—C8—C9	179.46 (12)	C6—C1—N1—C7	−6.65 (18)
C7—C8—C9—C10	0.7 (2)	C2—C1—N1—C7	174.37 (12)
C8—C9—C10—C11	0.4 (2)	C12—C7—N1—N2	−79.10 (16)
C9—C10—C11—C12	−1.0 (2)	C8—C7—N1—N2	100.30 (14)
C8—C7—C12—C11	0.5 (2)	C12—C7—N1—C1	109.40 (14)
N1—C7—C12—C11	179.92 (12)	C8—C7—N1—C1	−71.20 (16)
C10—C11—C12—C7	0.5 (2)	C14—C13—N2—N1	−178.95 (11)
N2—C13—C14—C15	−176.81 (14)	C1—N1—N2—C13	176.38 (12)
N2—C13—C14—O1	3.82 (19)	C7—N1—N2—C13	4.49 (18)
N2—C13—C14—O1	3.82 (19)	O2—O2—N3—O3	0.00 (9)
O1—C14—C15—C16	−0.05 (15)	O2—O2—N3—C23	0.00 (10)
O1—C14—C15—C16	−0.05 (15)	C22—C23—N3—O2	−125.38 (13)
C13—C14—C15—C16	−179.46 (13)	C18—C23—N3—O2	52.50 (16)
C14—C15—C16—C17	−0.12 (16)	C22—C23—N3—O2	−125.38 (13)
C15—C16—C17—O1	0.24 (15)	C18—C23—N3—O2	52.50 (16)
C15—C16—C17—O1	0.24 (15)	C22—C23—N3—O3	52.21 (16)
C15—C16—C17—C18	175.53 (14)	C18—C23—N3—O3	−129.90 (13)
C16—C17—C18—C19	−159.87 (15)	C15—C14—O1—O1	0.00 (10)
O1—C17—C18—C19	15.26 (17)	C13—C14—O1—O1	0.00 (11)
O1—C17—C18—C19	15.26 (17)	C15—C14—O1—C17	0.20 (14)
C16—C17—C18—C23	16.6 (2)	O1—C14—O1—C17	0E1 (4)
O1—C17—C18—C23	−168.24 (11)	C13—C14—O1—C17	179.68 (11)
O1—C17—C18—C23	−168.24 (11)	C16—C17—O1—O1	0.00 (8)
C23—C18—C19—C20	−0.53 (19)	C18—C17—O1—O1	0.00 (9)
C17—C18—C19—C20	176.29 (12)	C16—C17—O1—C14	−0.27 (14)
C18—C19—C20—C21	−0.8 (2)	O1—C17—O1—C14	0E1 (4)
C19—C20—C21—C22	1.0 (2)	C18—C17—O1—C14	−176.69 (10)

### Hydrogen-bond geometry (Å, º)

**Table:**

*D*—H···*A*	*D*—H	H···*A*	*D*···*A*	*D*—H···*A*
C8—H8···O2^i^	0.95	2.48	3.1294 (16)	126
C9—H9···O2^i^	0.95	2.69	3.2324 (18)	117
C11—H11···O3^ii^	0.95	2.57	3.4336 (18)	151
C12—H12···O3^iii^	0.95	2.48	3.3786 (18)	158
C16—H16···O2	0.95	2.56	2.9635 (17)	106
C19—H19···O1	0.95	2.39	2.7389 (16)	102

Symmetry codes: (i) *x*+1, *y*, *z*; (ii) −*x*+1/2, *y*−1/2, −*z*+1/2; (iii) *x*+1/2, *y*−1/2, *z*.
